# Kinetic Phase Diagrams of Ternary Al-Cu-Li System during Rapid Solidification: A Phase-Field Study

**DOI:** 10.3390/ma11020260

**Published:** 2018-02-08

**Authors:** Xiong Yang, Lijun Zhang, Sergey Sobolev, Yong Du

**Affiliations:** 1 State Key Laboratory of Powder Metallurgy, Central South University, Changsha 410083, China; youngfish1989@csu.edu.cn (X.Y.); yong-du@csu.edu.cn (Y.D.); 2 Institute of Problem of Chemical Physics, Russian Academy of Science, Moscow Region, Chernogolovka 142432, Russia; ssl55@yandex.ru

**Keywords:** Al-Cu-Li, rapid solidification, kinetic phase diagram, phase-field modeling

## Abstract

Kinetic phase diagrams in technical alloys at different solidification velocities during rapid solidification are of great importance for guiding the novel alloy preparation, but are usually absent due to extreme difficulty in performing experimental measurements. In this paper, a phase-field model with finite interface dissipation was employed to construct kinetic phase diagrams in the ternary Al-Cu-Li system for the first time. The time-elimination relaxation scheme was utilized. The solute trapping phenomenon during rapid solidification could be nicely described by the phase-field simulation, and the results obtained from the experiment measurement and/or the theoretical model were also well reproduced. Based on the predicted kinetic phase diagrams, it was found that with the increase of interface moving velocity and/or temperature, the gap between the liquidus and solidus gradually reduces, which illustrates the effect of solute trapping and tendency of diffusionless solidification.

## 1. Introduction

Aluminum-copper-lithium (Al-Cu-Li) alloys represent a promising new material in aerospace and defense industries due to their superior properties, such as low density and high specific strength [1,2]. However, one shortcoming of this type of alloy prepared using the traditional solidification technique lies in solute segregation towards the grain boundaries, resulting in formation of precipitates along grain boundaries and decreases of toughness of the alloys. Rapid solidification technology can be used as an important approach to reverse solute segregation during the preparation process, and thus create homogeneous alloys. The rapid extraction of thermal energy that occurs during rapid solidification process can provide large deviations from equilibrium, which helps to obtain non-equilibrium microstructures, such as amorphous and quasicrystals that show superior comprehensive properties compared with those in equilibrium or near equilibrium states [3,4]. Experimental results for alloys during rapid solidification show that under this non-equilibrium condition, solute concentration could differ significantly from that given by the equilibrium phase diagram. This effect in rapid solidification, known as “solute trapping” [3,5], has been studied extensively by experimental [6,7], theoretical [8,9,10,11,12,13,14,15,16] and numerical methods [17,18,19,20,21,22,23,24,25].

In the industrial design of new Al-Cu-Li alloys, the interface velocity-dependent kinetic phase diagram should serve as a useful tool to predict the phase concentrations and corresponding undercooling at the target solidification velocity. Aziz et al. [8] formulated a continuous growth model (CGM) by considering the flux balance across the moving solid-liquid interface, and predicted the velocity-dependent solute segregation coefficient *k*(*V*) at different interface moving velocities *V*s. By combining chemical rate theory, the CGM was employed to predict the kinetic interface condition diagram for ideal solution systems and binary Ag-Cu systems without/with solute drag effects [9]. Later on, Sobolev proposed a local-non-equilibrium model (LNM) [10,11,14,15,16], which took into account the relaxation to local equilibrium of the solute flux under rapid solidification conditions. The model predicted the abrupt transition to diffusionless and partitionless solidification with complete solute trapping when the interface velocity *V* reached the bulk liquid diffusion velocity *V_D_* [10,11,14,15,16]. The transition led also to coincidence of the effective (non-equilibrium) liquidus and solidus lines in kinetic phase diagram at *V* = *V_D_*. The results were in good agreement with the experimental data and molecular dynamic simulations [10,11,14,15,16]. 

Besides the above theoretical models, the phase-field approach is also a powerful numerical method for rapid solidification simulation and kinetic phase diagram prediction. Based on the well-known Wheeler-Boettinger-McFadden (WBM) model [17,18,19], Galenko and his co-workers developed a hyperbolic phase-field model, which introduced the diffusion flux term as the non-equilibrium contribution into the free energy functional. The model was applied to obtain the kinetic phase diagram in the binary Si-As system and predicted complete solute trapping at finite solidification velocity, which is in good agreement with the experimental results [21]. Recently, in the framework of the multi-phase-field (MPF) model [26,27,28], a phase-field model with finite interface dissipation has been developed to describe the non-equilibrium phase transformations [22,23]. In the model, the concentration field is split into the phase concentrations for the individual bulk phases. During the phase transition process, a local redistribution flux through the phase boundary is considered at the interface, and this solute exchange process between phases is described by a kinetic equation. The model has been applied to simulate the solute trapping in different binary alloys during rapid solidification [22,24,25] and later to predict the binary kinetic phase diagram [29].

Generally, the phase-field simulation of rapid solidification can be performed by setting initial alloy composition and system temperature as the input, and then solving temporal and spatial partial differential equations simultaneously by using the classic explicit scheme [22,23,25]. When the steady state is reached, the solute distribution and moving velocity of solid/liquid interface can be predicted. Generally, this standard explicit scheme is easily coded but inefficient to predict interface moving velocity dependent kinetic phase diagram. Thus, a different numerical scheme is proposed to eliminate the temporal variable in the evolution equations of phase field and concentration by introducing a reference of moving frame, *z* = *x* − *Vt*, which moves with a constant velocity *V* at the center of the interfacial region given by *ϕ* = 1/2 at *z* = 0 [20,21,29]. With the input of interface velocity and initial alloy composition, the steady-state concentration profile and temperature at the interface can be predicted via the relaxation resolution. Thus, with the input of the fixed interface velocity and varied initial alloy compositions, the corresponding interface velocity-dependent kinetic phase diagram can be obtained. Moreover, in the work of [20,21], the interface temperature was obtained by fixing the interface at origin point in the moving reference frame via a temperature relaxation equation, while in [29], the interface temperature is calculated via the Gibbs-Thomson equation directly and such numerical scheme is named as “the time-elimination relaxation scheme”. Additionally, it has been shown that with the equivalent input the time-elimination relaxation scheme and the standard explicit scheme can reach the unique solute distribution and temperature-velocity relationship in the length scale from nanometer to micrometer [29]. However, all the reported interface velocity-dependent kinetic phase diagrams in the literature are only limited to several simple binary systems [9,16,21,29]. It is of great theoretical and technical importance to explore ternary and even higher-order systems, which are much closer to the real materials.

Consequently, the phase-field model with finite interface dissipation together with the time-elimination relaxation scheme is to be utilized to predict the kinetic phase diagram in the ternary Al-Cu-Li system, as is the major task of this paper. Such kinetic phase diagrams can serve as the useful guidance for choosing the appropriate process parameters during preparation of novel Al-Cu-Li alloys using rapid solidification technology.

## 2. Phase-Field Model with Finite Interface Dissipation

According to references [22,23], a general system with an *α*-*β* transition and *i* = 1…*n* components can be described by the phase field *φ_α_*, which gives the local fractions of the phase *α* and its complement *φ_β_* = 1 − *φ_α_*, and the phase concentration fields ${\vec{c}}_{j}$ (*j* = *α* or *β*). The concentration vector ${\vec{c}}_{\alpha }$ is introduced to express the all phase concentration fields $c_{\alpha }^{i}$ in phase *α*. The overall concentration *c^i^* is given by a mixture rule varying with spatial variable *x* and time variable *t*:

(1) $$c^{i}\left(x,t\right)=\phi_{\alpha }\left(x,t\right)c_{\alpha }^{i}\left(x,t\right)+\phi_{\beta }\left(x,t\right)c_{\beta }^{i}\left(x,t\right)$$

The total free energy functional *F* consists of the interfacial part *f^intf^* and the chemical part *f^chem^*:

(2) $$F\left(\phi_{\alpha },{\vec{c}}_{\alpha },{\vec{c}}_{\beta }\right)=\int_{\Omega }\left\{f^{intf}\left(\phi_{\alpha }\right)+f^{chem}\left(\phi_{\alpha },{\vec{c}}_{\alpha },{\vec{c}}_{\beta }\right)\right\}d\Omega$$

The interfacial free energy density is defined by the gradients of the phase fields and the potential function:

(3) $$f^{intf}\left(\phi_{\alpha }\right)=\frac{4\sigma_{\alpha \beta }}{\eta }\left[-\frac{\eta^{2}}{\pi^{2}}\nabla \phi_{\alpha }\cdot \nabla \phi_{\beta }+\phi_{\alpha }\phi_{\beta }\right]$$

where *η* is the interface width, and *σ_αβ_* the interfacial energy between the *α* phase and *β* phase. The chemical free energy density functional is defined by:

(4) $$f^{chem}\left(\phi_{\alpha },{\vec{c}}_{\alpha },{\vec{c}}_{\beta }\right)=\phi_{\alpha }f_{\alpha }\left({\vec{c}}_{\alpha }\right)+\phi_{\beta }f_{\beta }\left({\vec{c}}_{\beta }\right)+\lambda^{i}\left[c^{i}-(\phi_{\alpha }c_{\alpha }^{i}+\phi_{\beta }c_{\beta }^{i})\right]$$

here, $f_{j}\left({\vec{c}}_{j}\right)$ is the volume free energy density of the individual phase *j*, which depends on the phase concentration ${\vec{c}}_{j}$ (*j* = *α* or *β*). If the molar volumes of *α* and *β* phases are assumed to be equal and independent of the concentration, i.e., $v_{m}^{\alpha }$ = $v_{m}^{\beta }$ = *v_m_*, the volume free energy densities can be linked to molar Gibbs free energy densities $g_{j}\left({\vec{c}}_{j}\right)$ (*j* = *α* or *β*), which can be provided by the thermodynamic databases:

(5) $$f_{j}\left({\vec{c}}_{j}\right)=\frac{1}{v_{m}}g_{j}\left({\vec{c}}_{j}\right),\;\left(j=\alpha \;\mathrm{or}\;\beta \right)$$

In Equation (4), the Lagrange parameters *λ^i^* is introduced to ensure the concentration conservation for component *i* ($c^{i}=\phi_{\alpha }c_{\alpha }^{i}+\phi_{\beta }c_{\beta }^{i}$), and the expressions are proposed as [23]:

(6) $$\lambda^{i}=\phi_{\alpha }\frac{\partial f_{\alpha }}{\partial c_{\alpha }^{i}}+\phi_{\beta }\frac{\partial f_{\beta }}{\partial c_{\beta }^{i}}-\frac{{\dot{\phi }}_{\alpha }c_{\alpha }^{i}+{\dot{\phi }}_{\beta }c_{\beta }^{i}}{P^{i}}$$

where *P^i^* has the dimension of an inverse action density (cm^3^/Js). In fact, *P^i^* is the rate constant of component *i* controlling the interface dissipation between phase *α* and *β*, which can be estimated as:

(7) $$P^{i}=\frac{8{\tilde{M}}^{i}}{\delta_{\mathrm{atom}}\eta }$$

where *δ*_atom_ is the atomic interface width, while ${\tilde{M}}^{i}$ is the atomic mobility for component *i* in the *α*-*β* interface as a mixture from the chemical mobility in each phase. Based on the above free energy functional, the evolution equations for phase concentrations can $c_{\alpha }^{i}$ be expressed as:

(8) ϕαc˙αi=∇(ϕα∑j=1n−1Dαijn∇cαj)+Piϕαϕβ(∂fβ∂cβi−∂fα∂cαi)+ϕαϕ˙α(cβi−cαi)

here, Dαijn is the chemical diffusivity in phase *α* with *n* as the dependent component, which can be experimentally measured or calculated from the atomic mobility databases. The second part in Equation (8) describes the flux of solute between phases due to the difference of the diffusion potential ${\tilde{\mu }}_{\alpha }^{i}=\partial f_{\alpha }/\partial c_{\alpha }^{i}$. The third term represents the change of the phase concentrations due to the phase field change ${\dot{\phi }}_{\alpha }$. As mentioned in reference [22], for fast exchange (large value of *P*), the model recovers the equilibrium phase transition process. In the case of small value of *P*, the non-equilibrium phase transition process, such as solute trapping, can be modeled.

According to Zhang et al. [23], the evolution equation for the phase field *φ_α_* is given by:

(9) $${\dot{\phi }}_{\alpha }=K_{\alpha \beta }\left\{\sigma_{\alpha \beta }\left[\nabla^{2}\phi_{\alpha }+\frac{\pi^{2}}{\eta^{2}}(\phi_{\alpha }-\frac{1}{2})\right]+\frac{\pi }{\eta }\sqrt{\phi_{\alpha }(1-\phi_{\alpha })}\Delta g_{\alpha \beta }^{phi}\right\}$$

where the chemical driving force, $\Delta g_{\alpha \beta }^{phi}$, is expressed as:

(10) $$\Delta g_{\alpha \beta }^{phi}=f_{\beta }-f_{\alpha }-\sum_{i=1}^{n-1}\left(\phi_{\alpha }\frac{\partial f_{\alpha }}{\partial c_{\alpha }^{i}}+\phi_{\beta }\frac{\partial f_{\beta }}{\partial c_{\beta }^{i}}\right)(c_{\beta }^{i}-c_{\alpha }^{i})$$

*K_αβ_* in Equation (9) is the modified interface mobility between phase *α* and *β*, which is given by:

(11) $$K_{\alpha \beta }=\frac{8\eta \mu_{\alpha \beta }}{8\eta +\mu_{\alpha \beta }\pi^{2}\sum_{i=1}^{n-1}\frac{{\left(c_{\alpha }^{i}-c_{\beta }^{i}\right)}^{2}}{P_{\alpha \beta }^{i}}}$$

where *μ_αβ_* is the physical interface mobility between *α* and *β* phase.

## 3. Time-Elimination Relaxation Scheme

For a given system with only a *α*-*β* transition, the time-elimination relaxation scheme can be applied [29], which is started by introducing a reference of moving frame:

(12) z=x−Vt

propagating at a constant velocity *V* and coincident with the center of the interface given by *φ_α_* = 1/2 at *z* = 0. Equations (8) and (9) then become:

(13) −Vϕα∂cαi∂z=∂∂z(ϕα∑j=1n−1Dαijn∂cαj∂z)+Pαβiϕαϕβ(∂fβ∂cβi−∂fα∂cαi)−Vϕα(cβi−cαi)∂ϕα∂z

(14) $$-V\frac{\partial \phi_{\alpha }}{\partial z}=K_{\alpha \beta }\left\{\sigma_{\alpha \beta }\left[\frac{\partial^{2}\phi_{\alpha }}{\partial z^{2}}+\frac{\pi^{2}}{\eta^{2}}\left(\phi_{\alpha }-\frac{1}{2}\right)\right]+\frac{\pi }{\eta }\sqrt{\phi_{\alpha }(1-\phi_{\alpha })}\Delta g_{\alpha \beta }^{phi}\right\}$$

The simulation temperature is resolved by the Gibbs-Thomson equation:

(15) $$V=\mu_{\alpha \beta }\left(\sigma \kappa +\Delta g_{\alpha \beta }^{phi}\right)$$

where *κ* is the curvature term, while $\Delta g_{\alpha \beta }^{phi}$ is the chemical driving force which depends on the simulation temperature and compositions. In 1-D steady-state simulation, one can have the curvature *κ* = 0 and *K_αβ_* in Equation (14) is used to describe the influence of finite diffusion and redistribution on the phase transition process. Thus, Equation (15) can be simplified as:

(16) $$V=K_{\alpha \beta }\cdot \Delta g_{\alpha \beta }^{phi}$$

## 4. Results and Discussion

The Al-Cu-Li ternary system and two sub-binary systems (i.e., Al-Cu and Al-Li) in the Al-rich corner were chosen as the target in the present work. The molar Gibbs energy densities of the liquid and solid phases in the binary Al-Cu system were taken from the COST 507 report [30], while those in the binary Al-Li system were from the work of Hallstedt et al. [31]. For the ternary Al-Cu-Li system, the optimized interaction parameters in Moser et al. [32] were utilized. For Al-Cu system, the interdiffusion coefficients in solid ($D_{S}^{\mathrm{Al}-\mathrm{Cu}}$) and liquid ($D_{L}^{\mathrm{Al}-\mathrm{Cu}}$) phases were fixed as 2.51 × 10^−9^ cm^2^/s [33] and 4.45 × 10^−5^ cm^2^/s [7], respectively, while $D_{S}^{\mathrm{Al}-\mathrm{Li}}$ and $D_{L}^{\mathrm{Al}-\mathrm{Li}}$ were respectively fixed as 3.95 × 10^−9^ cm^2^/s [34] and 8.61 × 10^−5^ cm^2^/s [35] for the Al-Li system. Due to lack of any kinetic information on the ternary Al-Cu-Li system, the diffusivities of binary Al-Cu and Al-Li systems were directly used as the diagonal interdiffusion coefficients in the ternary Al-Cu-Li, and all off-diagonal interdiffusion coefficients were ignored for simplification, i.e., DSCuCuAl=DSAl-Cu, DSLiLiAl=DSAl-Li, DLCuCuAl=DLAl-Cu, DLLiLiAl=DLAl-Li, DijkAl=0 (*i* = *S* or *L* and *j* ≠ *k*). The interface mobility, *μ_LS_*, was chosen as 2.46 cm^4^/Js in order to match the kinetic relationship between the undercooling and the solidification velocity during the rapid solidification [25]. The initial concentrations of alloys are set as Al-1.1 at. % Cu, Al-15 at. % Li and Al-1.1 at. % Cu-15 at. % Li, respectively. The grid size *∆x* is 1.25 × 10^−^^7^ cm and the total simulation length was 6.25 μm (5000*∆x*). All the numerical and thermophysical parameters used for the simulation are listed in Table 1. The phase-field simulations using the time-elimination relaxation scheme were conducted by solving the concentration evolution equations (i.e., Equation (13)), the phase-field evolution equation (i.e., Equation (14)) and Equation (16). The left and right boundaries for phase field were set as insulation conditions. As for the concentrations, an insulation condition was used for the left boundary, while the concentration at the right boundary was fixed at the initial alloy concentration. During the phase-field simulation, the interface moving velocity was fixed, while the solidification temperature and composition distribution were varied. The phase-field simulation program was coded by using C++, and the 1-D phase-field simulation was then performed. Because the time-elimination scheme was used for the phase-field simulation, the inputs include initial concentrations and interface moving velocity, while the outputs resulting from the simulation are solidification temperature and composition distribution.

### 4.1. Al-Cu and Al-Li Binary Systems

**Figure 1:**
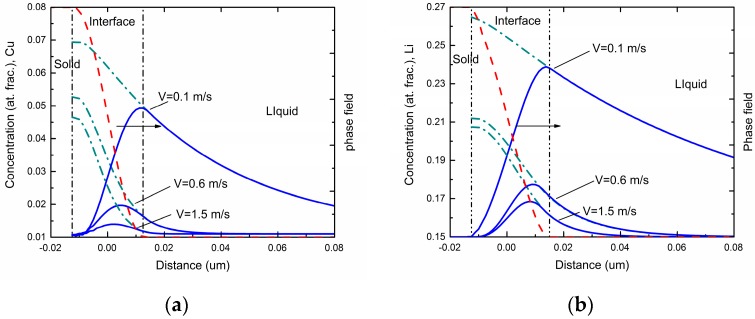
Phase-field simulated steady-state concentration profiles with three different interface moving velocities in: (**a**) Al-Cu system, (**b**) Al-Li system. The solid lines denote the overall concentrations, while the dotted lines denote the liquid concentrations.

Figure 1 shows the steady-state concentration profiles via the phase-field simulations in different interface moving velocities for Al-Cu and Al-Li alloys respectively. As can be seen in the figures, the overall concentration profile in the solid phase has a uniform value equal to the far-field concentration *c*_0_ in the liquid under the steady state. The overall concentration increases in the interface region due to the rejection of solute atoms by the growing solid. In the liquid ahead of the interface, a concentration boundary layer forms due to the diffusive transport of the rejected solute atoms into the liquid. As interface velocity increases, both maximum solute concentration and spatial penetration of the liquid concentration profile diminishes, which demonstrates a decrease of solute segregation at the interface and an occurrence of the solute trapping.

Generally, solute trapping effect can be characterized by velocity-dependent solute segregation coefficients *k^i^*(*V*). In the phase-field model with finite interface dissipation, the individual phase concentrations are utilized, and each position over the interface can be assumed to be a sharp interface. Thus, the solute segregation coefficient for component *i* is defined as [22]:

(17) $$k^{i}\left(V\right)=\frac{\mathrm{far}-\mathrm{field}\;\mathrm{concentration}}{\mathrm{maximun}\;\mathrm{of}\;\mathrm{the}\;\mathrm{liquid}\;\mathrm{concentration}}=\frac{c_{S}^{i}}{max\left[c_{L}^{i}\right]}=\frac{c_{S}^{i}}{c_{L}^{i}\left(\phi =0.9999\right)}$$

**Figure 2:**
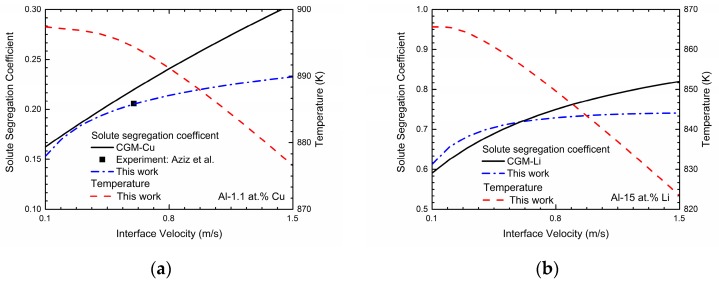
Phase-field simulated solute segregation coefficient and solidification temperature as a function of interface velocity in: (**a**) Al-1.1 at. % Cu alloy and (**b**) Al-15 at. % Li alloy.

The far-field concentration in the liquid is equal to the concentration in the bulk solid under the steady-state condition, and the maximum of the liquid concentration across the interface is at the position adjacent to the solid bulk region. For simplicity, the maximum of the liquid concentration is assumed to be the liquid concentration at *φ* = 0.9999. These concentrations can be achieved in a typical steady-state concentration profile during rapid solidification. The simulated velocity-dependent solute segregation coefficients *k*(*V*) of Al-Cu and Al-Li system are exhibited in Figure 2. As shown in the figure, the predicted velocity-dependent solute segregation coefficients *k*(*V*) generally increases as interface velocity increases for both two binary systems, indicating the enhancement of the solute-trapping effect. According to [22], the decrease in the interface permeability *P* can significantly enhance the solute-trapping effect, which owes to the decrease of the chemical partitioning process. Thus, the intended solute segregation coefficients can be easily obtained by adjusting *P*. For Al-Cu system, the interface permeability *P* is set to be 1.32 × 10^4^ cm^3^/Js to reproduce the experimental data from Aziz et al. [7]. As for the Al-Li system, due to the lack of experimental data, the continuous growth model (CGM) [8,9] is applied to estimate the interface permeability *P*:

(18) $$k\left(V\right)=\frac{V+V_{D}\cdot k_{e}}{V+V_{D}}$$

where *V_D_* is the diffusive speed of solute atom and *k_e_* is the equilibrium partition coefficient. According to [38], the diffusive speed is estimated as 1 m/s and equilibrium partition coefficient of lithium within aluminum is calculated as 0.55. Thus, *P* is set as 1.63 × 10^4^ cm^3^/Js and the *k*(*V*) curves simulated via this work and predicted by CGM are both presented in the Figure 2b. One point that should be paid attention is that when the solidification velocity *V* increases approaching to the diffusive speed of solute atom *V_D_*, the local non-equilibrium model (LNM) is more appropriate, which shows the abrupt change of *k*(*V*) to unit at finite solidification velocity *V* = *V_D_* [10,11,14,15,16]:

(19) $$k\left(V\right)=\left\{ \begin{array}{ll} \frac{k_{e}\left(1-V^{2}/V_{D}^{2}\right)+V/V_{D}}{\left(1-V^{2}/V_{D}^{2}\right)+V/V_{D}}, & V<V_{D}\\ 1, & V\geq V_{D} \end{array}\right.$$

However, due to the fact that local non-equilibrium effect of the diffusion solute has not been considered in the present phase-field model, we only utilize the results from CGM to predict the interface permeability *P* for this work. Figure 2 also exhibits the relations between the interface velocity *V* and the solidification temperature *T* in Al-Cu and Al-Li system. As shown in the figures, the solidification temperature *T* decreases monotonically as the interface velocity *V* increases, which demonstrates the increasing undercooling needed to obtain the certain interface velocity.

**Figure 3:**
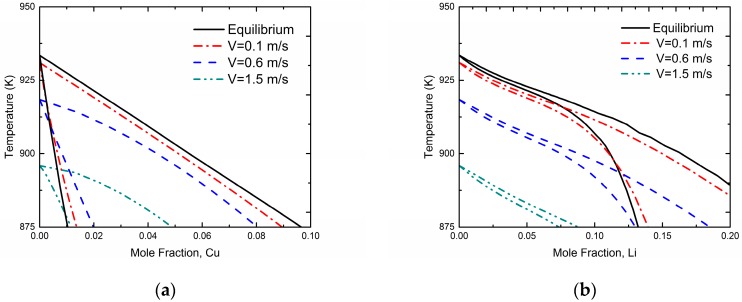
Model-predicted kinetic phase diagrams at different interface velocities due to the 1-D phase-field simulation using the time-elimination relaxation scheme of the (**a**) Al-Cu system, (**b**) Al-Li system. Solid lines represent the equilibrium phase diagram of the Si-As system.

Figure 3 demonstrates kinetic phase diagrams at different interface velocities based on the present phase-field simulation for Al-Cu and Al-Li systems, respectively. For comparison, the equilibrium phase diagram of the Al-Cu and Al-Li systems (i.e., *V* approaches to 0 m/s, denoted by solid lines) are also shown in Figure 3. The results show that the gap between liquidus and solidus gradually reduces as the interface moving velocity *V* increases, which demonstrates an occurrence of solute trapping and the tendency to diffusionless solidification. In addition, the “kinetic melting point” of pure Al, which is actually the temperature to provide the undercooling for the certain solidification velocity, can be determined by the Gibbs-Thomson equation directly:

(20) $$V=\mu_{LS}\cdot \Delta G_{L\to S}^{\mathrm{Al}}\left(T\right)$$

where *μ_LS_* is the liquid-solid interface mobility and $\Delta G_{L\to S}^{\mathrm{Al}}\left(T\right)$ is the molar free energy difference between liquid and solid phase for pure Al at certain temperature *T*. It can be seen from the Figure 3 that this “kinetic melting point” also reduces as interface velocity increases.

### 4.2. Al-Cu-Li Ternary System

**Figure 4:**
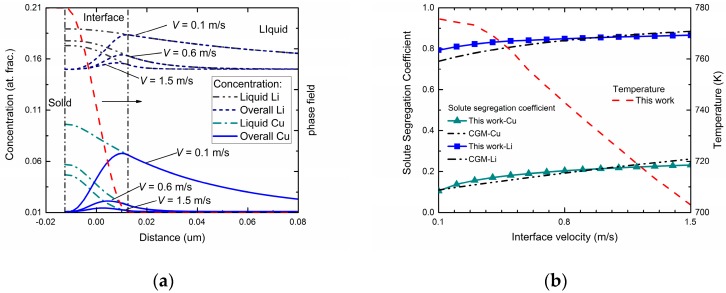
(**a**) Phase-field simulated steady-state concentration profiles with three different interface moving velocities in the Al-Cu-Li system. (**b**) Phase-field simulated solute segregation coefficient and solidification temperature as a function of interface velocity in Al-Cu-Li system.

Figure 4a exhibits the phase-field simulated steady-state concentration profiles in three different interface moving velocities for Al-Cu-Li ternary alloys. As can be seen in the figures, the overall concentration profile and liquid concentration profile show similar tendencies compared with the two binary systems. Besides, diffusionless solidification trends to happen as the interface velocity increases. However, compared with the cases of binary alloys, the newly added component significantly changed the concentration distribution of original component at a certain interface moving velocity. For the Cu component, due to the interact of the Li atoms, a decreasing tendency of diffusionless solidification was observed. By comparison, the composition distribution of Li component showed a reduced fluctuation in the interface region under the influence of Cu atoms, which demonstrates an increasing tendency of solute trapping. Figure 4b shows the velocity-dependent solute segregation coefficient for both Cu and Li atoms via this phase-field simulation. To determine the interface permeability parameters *P*^Cu^ and *P*^Li^, the *k*_Cu_(*V*) and *k*_Li_(*V*) profiles were adjusted to fit the curves predicted by CGM. Similar to the predicted k(V) of the two binary systems shown in Figure 2, velocity-dependent *k*_Cu_(*V*) and *k*_Li_(*V*) both increased as interface velocity increased, indicating a tendency to diffusionless solidification. Meanwhile, compared with the results from the binary systems, the solute trapping effect for Li component is enhanced, while for the Cu component it is weakened. The temperature-velocity profile is also exhibited in Figure 4b. Compared with the temperature-velocity curves in Figure 2, the same monotonicity of *T*-*V* profile was shown while a much lower temperature was needed to provide the same undercooling and interface moving velocity.

**Figure 5:**
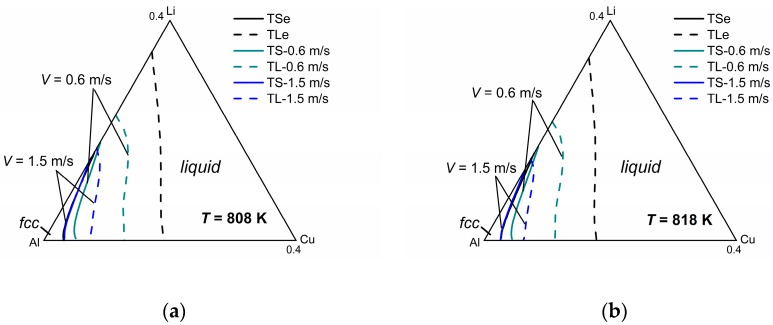

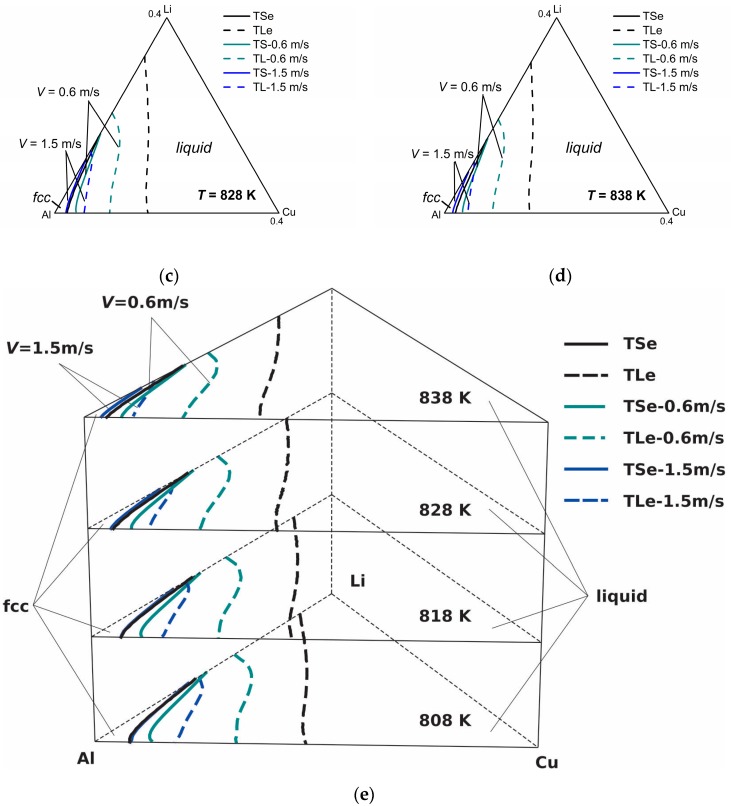
Model-predicted ternary Al-Cu-Li isothermal sections with different solidificaiton velocities at (**a**) 808 K, (**b**) 818 K, (**c**) 828 K, and (**d**) 838 K, as well as (**e**) the three-dimensional (3-D) Al-Cu-Li phase diagram.

Figure 5 shows the model-predicted isothermal section diagrams of the ternary Al-Cu-Li system at four different temperatures (808 K, 818 K, 828 K, and 838 K) under interface moving velocities of 0.6 m/s and 1.5 m/s, from which the three-dimensional (3-D) phase diagram (show in Figure 5e) can be established in a straightforward way. The liquidus and solidus of *V* = 0.1 m/s are not presented in this figure for the reason that they almost coincide with the equilibrium liquidus and solidus. It can be seen from the isothermal sections that the solid-liquid phase region diminishes as the solidification velocity increases at a specific temperature, which illustrates the tendency to diffusionless solidification. Moreover, it can be clearly seen from the 3-D phase diagram that as temperature increases, the gap between the liquidus and solidus lines shrinks for the same interface moving velocity, indicating the enhancement of the solute trapping phenomenon. Thus, for the target non-equilibrium alloy compositions, such kinetic diagrams can give the required solidification velocity needed for the production industry.

## 5. Conclusions

The phase-field simulation was performed to predict interface velocity-dependent solidification kinetic phase diagram in Al-Cu-Li ternary alloy using the time-elimination relaxation scheme in the framework of the phase-field model with finite interface dissipation. By adjusting the kinetic parameter interface permeability *P*, the solute trapping phenomenon can be nicely described and the results obtained from experiment or theoretical model can be well reproduced. Similar to the previously predicted binary kinetic phase diagrams via theoretical models and numerical methods, the solidification ternary kinetic phase diagrams show the gradual reduction of the gap between the liquidus and solidus as the interface moving velocity and temperature increase, which illustrates the effect of solute trapping and tendency of diffusionless solidification. Meanwhile, different from the binary alloys, the composition distribution of the components at certain interface moving velocities could obviously change, due to the effect of the third component. It is anticipated that such kinetic phase diagrams at different solidification velocities and temperatures are of great importance for guiding the preparation of novel Al-Cu-Li alloys using rapid solidification technology.

## Figures and Tables

**Table 1:** Materials parameters for the phase-field simulation of rapid solidification in Al-Cu-Li alloy

Parameter	Symbol	Value	Reference
Grid spacing	*∆x*	1.25 × 10^−^^7^ cm	present work
Simulatoin length	*L*	6.25 × 10^−^^4^ cm	present work
Interface width	*η*	2.5 × 10^−^^6^ cm	present work
Interfacial energy	*σ_LS_*	1.06 × 10^−5^ J/cm^2^	[36]
Interface mobility	*μ_LS_*	2.46 cm^4^/Js	[25]
Diffusivity of solid in Al-Cu	$D_{S}^{\mathrm{Al}-\mathrm{Cu}}$	2.51 × 10^−9^ cm^2^/s	[33]
Diffusivity of liquid in Al-Cu	$D_{L}^{\mathrm{Al}-\mathrm{Cu}}$	4.45 × 10^−5^ cm^2^/s	[7]
Diffusivity of solid in Al-Li	$D_{S}^{\mathrm{Al}-\mathrm{Li}}$	3.95 × 10^−9^ cm^2^/s	[34]
Diffusivity of liquid in Al-Li	$D_{L}^{\mathrm{Al}-\mathrm{Li}}$	8.61 × 10^−5^ cm^2^/s	[35]
Melting temperature of pure Al	*T_m_*	933.47 K	[37]
Molar volume	*V_m_*	11 cm^3^/mol	present work
